# CRISPR/Cas9-mediated editing of *SlALS1* enhances fruit sweetness in tomato

**DOI:** 10.3389/fpls.2026.1927332

**Published:** 2026-10-08

**Authors:** So Hee Yang, Soon-Ki Han, Yeonjong Koo

**Affiliations:** 1 Department of Agricultural Chemistry, Chonnam National University, Gwangju, Republic of Korea; 2 Department of Biological Sciences, Ajou University, Suwon, Republic of Korea; 3 Institute of Environmentally-friendly Agriculture, Chonnam National University, Gwangju, Republic of Korea; 4 Institute of Plant Biomaterials and Biomedical Science, Chonnam National University, Gwangju, Republic of Korea

**Keywords:** acetolactate synthase 1 (SlALS1), branched-chain amino acid biosynthesis, CRISPR/Cas9, fruit sweetness, precision breeding, solanum lycopersicum, soluble sugar accumulation, tomato

## Abstract

Fruit sweetness is a primary determinant of consumer preference and market value in tomato (*Solanum lycopersicum*), yet increasing sugar content without negatively affecting plant performance remains a major challenge in breeding programs. In this study, we targeted *Acetolactate synthase 1* (*SlALS1*), a key enzyme in branched-chain amino acids (BCAAs) biosynthesis, to investigate whether modulation of primary metabolism could enhance fruit sugar accumulation. Tomato contains two ALS homologs, *SlALS1* and *SlALS2*. Using CRISPR/Cas9-mediated genome editing, we generated *SlALS1* knock-out lines in the Micro-Tom background. Loss of *SlALS1* function led to a significant reduction in the levels of valine, leucine, and isoleucine in fruit tissues, confirming disruption of the BCAA biosynthetic pathway. Notably, soluble sugar content was markedly increased in the mutant lines, with glucose and fructose levels elevated by approximately 1.3-fold compared with wild-type fruits, resulting in a significantly higher Brix index. Metabolic profiling indicates that reduced BCAA biosynthesis may influence carbon allocation toward soluble sugar accumulation in fruit tissues, revealing an association between amino acid metabolism and carbohydrate accumulation during fruit development. Our findings demonstrate that precise editing of a central metabolic enzyme can enhance tomato fruit sweetness and suggest that *SlALS1* may represent a promising target for genome editing aimed at improving fruit quality traits. However, further evaluation of agronomic traits, including plant growth and fruit yield, will be necessary to assess its potential for practical breeding applications.

## Introduction

Tomato (*Solanum lycopersicum*) is one of the most widely cultivated horticultural crops worldwide and is extensively utilized in fresh consumption, cooking, and processed food products (Quinet et al., 2019; Dorais et al., 2008). Although botanically classified as a berry, tomato is commonly regarded as a vegetable because of its culinary applications (The Editors of Encyclopaedia Britannica, 2026). In many countries, including Korea, tomatoes are primarily consumed fresh, and consumers generally prefer fruits with enhanced sweetness and distinctive flavor profiles (Kaur et al., 2023). Fruit quality and taste are largely determined by sugar content, which depends on the accumulation of soluble sugars such as glucose, fructose, and sucrose (Beckles et al., 2012; Ahiakpa et al., 2021). Glucose serves as a major energy source in plant metabolism (Kleczkowski et al., 2010), whereas fructose contributes a stronger sweet taste perception. Sucrose, a disaccharide composed of glucose and fructose, also plays an important role in determining fruit sweetness and overall flavor quality (Balibrea et al., 2006). Therefore, the balance and interaction among these sugars are critical factors influencing tomato flavor and consumer preference (Luo et al., 2023).

Considerable efforts have been made to enhance tomato sweetness through various approaches. One commercially utilized method involves the application of sweeteners such as stevia, which can increase fruit sweetness from approximately 6°Bx to as high as 12°Bx (Lazou et al., 2020). Such high-sugar tomatoes command premium market prices due to their improved taste. In addition, several high-sugar tomato cultivars developed through conventional breeding, including Apple tomato, Honey tomato, and Daejeo tomato, exhibit soluble solids contents of approximately 9–10°Bx (Park et al., 2015). Similarly, the cultivars Sugar Drop, developed for the UK market, and Honey Bombs, commercialized in Canada, exhibit sugar contents ranging from 9–13°Bx and 9–12°Bx, respectively. These cultivars are highly valued by consumers because of their superior sweetness and flavor characteristics.

Among the factors affecting fruit quality, amino acid metabolism has been recognized as an important determinant of tomato flavor development (Lu and Zhu, 2022). Amino acids function as precursors of volatile compounds responsible for aroma formation (Schwab et al., 2008) and are essential components of protein biosynthesis (Lopez and Mohiuddin, 2024). Through various metabolic pathways, amino acids are converted into volatile aldehydes, esters, and other compounds that contribute to the characteristic aroma and flavor of ripe tomato fruits (Kaur et al., 2023). For example, glutamate and aspartate are associated with umami perception and contribute significantly to the savory taste of tomatoes (Alfosea-Simón et al., 2021; Zhang et al., 2015; Sorrequieta et al., 2010). Changes in amino acid composition during fruit ripening therefore have substantial effects on overall fruit flavor and quality.

*Acetolactate synthase (ALS)*, also known as *acetohydroxyacid synthase (AHAS)*, is a key enzyme involved in amino acid biosynthesis (Kochevenko and Fernie, 2011). ALS catalyzes the first committed step in the synthesis of BCAAs, including valine, leucine, and isoleucine, by converting pyruvate into precursor molecules such as acetolactate (Yang et al., 2022; McCourt and Duggleby, 2006). Because ALS occupies a central position linking amino acid biosynthesis and carbon metabolism, alterations in ALS activity may influence both amino acid accumulation and carbohydrate metabolism (Nie et al., 2018). However, it remains unclear whether modification or suppression of ALS activity can alter amino acid composition and sugar accumulation in tomato fruits, thereby affecting fruit sweetness. Understanding the regulatory role of ALS may provide valuable genetic insights for the development of high-sugar tomato cultivars.

Recent advances in genome-editing technologies have provided powerful tools for functional genomics and crop improvement (Abdallah et al., 2015; Yang et al., 2024). Among these technologies, the CRISPR-Cas9 system has emerged as a highly efficient and precise platform for targeted genome modification, significantly accelerating crop breeding programs (Pan et al., 2016). Owing to its versatility and effectiveness, CRISPR-Cas9 has been widely adopted for gene function analysis and trait improvement in tomato and other crop species (Xu et al., 2020; Li et al., 2018; Nie et al., 2024).

The primary objective of this study was to develop high-sugar tomato lines through CRISPR-Cas9-mediated editing of the *SlALS1* gene. We further investigated the role of *SlALS1* in regulating amino acid accumulation and sugar metabolism in tomato fruits, aiming to provide new insights into the molecular mechanisms underlying fruit sweetness. In addition, the ALS inhibitor imazethapyr was employed to examine the metabolic consequences of ALS suppression in tomato leaves and fruits (Zabalza et al., 2013). By integrating genetic and chemical approaches, this study seeks to elucidate the relationship between ALS activity and sugar accumulation, thereby providing a novel strategy for improving tomato flavor and fruit quality.

## Materials and methods

### gRNA design and construction of CRISPR/Cas9 vectors

Guide RNAs (gRNAs) for genome editing were designed using the CRISPR-P 2.0 program (http://crispr.hzau.edu.cn/cgi-bin/CRISPR2/CRISPR). Candidate target sites were selected by identifying 20-bp sequences immediately upstream of the CRISPR/Cas9 protospacer adjacent motif (PAM; 5′-NGG) within the target genes in the tomato genome. gRNAs with high predicted on-target specificity and minimal off-target activity were selected for subsequent experiments. For *SlALS1*, the oligonucleotides 5′-GATTGATTTGGGAATGGCGACACAG-3′ and 5′-AAACCTGTGTCGCCATTCCCAAATC-3′ were annealed and cloned into the Esp3I-digested pYPQ141A vector (Lowder et al., 2015). Similarly, for *SlALS2*, the oligonucleotides 5′-GATTGACTTGGGAATGGTGGTTCAA-3′ and 5′-AAACTTGAACCACCATTCCCAAGTC-3′ were annealed and inserted into the same vector. The resulting pYPQ141A-gRNA entry clones and the Cas9-containing pYPQ154 vector (Lowder et al., 2015) were recombined into the plant transformation vector pMDC32 through an LR recombination reaction (Yang et al., 2022). In this construct, gRNA expression was driven by the *Arabidopsis U6* promoter, while the *Cas9* gene was expressed under the control of the Cauliflower mosaic virus (CaMV) 35S promoter. The pMDC32 vector contains a kanamycin resistance gene for bacterial selection and a hygromycin resistance gene for plant selection. The final CRISPR/Cas9 construct was introduced into *Agrobacterium tumefaciens* strain GV3101 and subsequently used for transient expression assays in tomato cotyledons and for stable genetic transformation of tomato plants.

### Tomato seed germination

Tomato (*Solanum lycopersicum* cv. Micro-Tom) seeds were surface-sterilized with 10% sodium hypochlorite (NaClO) and sown on 3S medium (Duchefa Biochemie, Haarlem, Netherlands). The seeds were subjected to cold treatment at 4 °C for 5 days to promote germination and subsequently grown at 25 °C under a 12 h light/12 h dark photoperiod with a light intensity of 95 μmol m^-^² s^-^¹.

### Transient expression of the CRISPR/Cas9 system in tomato cotyledons

To evaluate the functionality and editing efficiency of the designed gRNAs, Agrobacterium-mediated transient expression assays were performed in tomato cotyledons. Agrobacterium harboring the CRISPR/Cas9 constructs were grown in LB medium supplemented with kanamycin, rifampicin, and gentamicin (50 μg mL^-^¹ each) at 28 °C for 24 h. Subsequently, 10% (v/v) of the starter culture was inoculated into fresh LB medium and incubated at 28 °C until the optical density at 600 nm (OD_600_) reached 0.8.

One milliliter of bacterial culture was collected by centrifugation at 7,000 rpm for 1 min and resuspended in infiltration buffer consisting of distilled water (50 mL), MS vitamins (0.05 g), sucrose (0.37 g), 1 mM IAA (12.5 μL), and 10 mM zeatin (12.5 μL). The suspension was incubated at 28 °C for 3 h prior to infiltration. Approximately 1 mL of the Agrobacterium suspension was infiltrated into the abaxial side of cotyledons excised from 10–15-day-old tomato seedlings grown as described above using a needleless syringe. The infiltrated cotyledons were maintained in a growth chamber at 24 °C for 5 days before further analysis.

### Agrobacterium-mediated transformation of tomato

Cotyledon explants were excised from 10–14-day-old tomato seedlings grown as described above and cut into halves prior to transformation. The explants were immersed for 5 min in an Agrobacterium suspension harboring the CRISPR/Cas9 expression vector. After infection, the explants were placed on co-cultivation medium (COM: MS salts, MS vitamins, 8 g L^-^¹ plant agar, 30 g L^-^¹ sucrose, 0.1 mg L^-^¹ IAA, 10 μM zeatin, and 200 μM acetosyringone, pH 5.8) with the adaxial side facing upward and incubated in the dark at 25 °C for 1 day.

Following co-cultivation, the infected explants were transferred to shoot regeneration medium (SRM: MS salts, MS vitamins, 8 g L^-^¹ plant agar, 30 g L^-^¹ sucrose, 250 mg L^-^¹ cefotaxime, and 50 mg L^-^¹ hygromycin, pH 5.8) and cultured at 25 °C until callus formation. Callus resistant to hygromycin was subsequently transferred to fresh SRM containing 50 mg L^-^¹ hygromycin for the selection of transformants and shoot regeneration. Explants were subcultured onto fresh SRM every two weeks for a total of four passages.

Regenerated shoots were excised and transferred to rooting medium (RM: MS salts, MS vitamins, 8 g L^-^¹ plant agar, 15 g L^-^¹ sucrose, 1 mg L^-^¹ IBA, 250 mg L^-^¹ cefotaxime, and 50 mg L^-^¹ hygromycin, pH 5.8) to induce root development. Independent transgenic plants with well-developed root systems were transferred to soil for acclimatization.

### Greenhouse growth conditions

Wild-type and transgenic tomato plants were grown simultaneously in a greenhouse at Chonnam National University, Gwangju, Republic of Korea, during the 2024 growing season. Plants were maintained at 25 ± 2 °C under a 12 h light/12 h dark photoperiod with supplemental LED lighting. Plants were irrigated as needed without additional fertilizer application. Fruits from both wild-type and transgenic plants were harvested for subsequent analyses.

### Genomic DNA extraction and genome editing analysis

Genomic DNA was extracted from plant tissues using a rapid extraction method as previously described (Edwards et al., 1991). To determine the genotypes of CRISPR/Cas9-edited ALS alleles, the target region was amplified by PCR using genomic DNA as a template. The primers used for amplification were the forward primer (5′-TGTGGCGAGTACTTTGATGGGACT-3′) and reverse primer (5′-GCACCATTACTGGGAATCATAGGC-3′). The resulting PCR amplicons were subjected to next-generation sequencing (NGS). Approximately 20,000–100,000 sequencing reads were obtained for each sample. The sequencing data were analyzed using CRISPR RGEN Tools to identify and quantify genome-editing events, including insertions, deletions, and sequence substitutions within the target region.

### Analysis of carbohydrates, amino acids, and organic acids in tomato fruits

Tomato fruits were collected from three independent plants per genotype at the red ripening stage. Five to six fruits from each plant were individually harvested and analyzed separately. The average value obtained from fruits of each plant was considered as one biological replicate. For metabolite analysis, fruits were homogenized using a tissue grinder. The homogenates were centrifuged at 1,000 × g for 2 min, and the supernatants were filtered through a 0.2 μm of membrane filter.

Glucose, fructose, and sucrose concentrations were quantified using high-performance liquid chromatography (HPLC; Dionex Ultimate 3000, Thermo Dionex, Germany). Sugars were separated on a Sugar-Pak column (300 × 6.5 mm; Waters, USA) using distilled water as the mobile phase. The chromatographic conditions were a column temperature of 70 °C, a flow rate of 0.5 mL min^-^¹, and an injection volume of 10 μL. Sugars were detected using a Shodex RI-101 refractive index detector (Shodex, Japan). Sugar concentrations were quantified by comparing chromatographic peak areas with external calibration curves generated using authentic glucose, fructose, and sucrose standards.

Amino acids were extracted from tomato fruit homogenates using 75% ethanol. The extracts were subjected to ultrasonic extraction for 1 h, followed by shaking at room temperature for 1 h. The extracts were filtered through a 0.2 μm of membrane filter prior to analysis. Amino acids and amino acid-related metabolites were analyzed using an HPLC system (Dionex Ultimate 3000, Thermo Dionex, Germany) equipped with an Inno C18 column (4.6 × 150 mm, 5 μm, Younjin Biochrom, Korea). Precolumn derivatization with o-phthalaldehyde (OPA) and 9-fluorenylmethyl chloroformate (FMOC) was performed automatically by the autosampler according to the manufacturer’s protocol (Agilent Technologies). Separation was carried out using 40 mM sodium phosphate buffer (pH 7.0) as mobile phase A and distilled water/acetonitrile/methanol (10:45:45, v/v/v) as mobile phase B. The gradient program was as follows: 95% A/5% B at 0–3 min, 45% A/55% B at 24 min, 20% A/80% B at 25–31 min, and 95% A/5% B at 34.5–35 min. The flow rate was maintained at 1.5 mL/min throughout the analysis. The column temperature was maintained at 40 °C, and the injection volume was 0.5 μL. Amino acids were detected using fluorescence detection (excitation/emission: 340/450 nm for OPA derivatives and 266/305 nm for FMOC derivatives) and UV detection at 338 nm. Amino acid concentrations were quantified by comparison of chromatographic peak areas with external calibration curves generated using authentic amino acid standards. Chromatographic data were processed using Chromeleon version 6.8 software.

Organic acids, including citric and malic acids, were analyzed using an HPLC system (Ultimate 3000, Thermo Dionex, USA) equipped with an Aminex 87H column (300 × 10 mm, Bio-Rad, USA). The mobile phase was 0.01 N sulfuric acid (H_2_SO_4_), with a flow rate of 0.5 mL min^-^¹. The column oven temperature was maintained at 40 °C, and the injection volume was 10 μL. Organic acids were detected using a refractive index detector (RefractoMAX520, ERC, Japan) and UV detection at 210 nm. The concentrations of citric and malic acids were quantified by comparison of chromatographic peak areas with external calibration curves generated using authentic standards.

### Quantitative real-time PCR (RT-qPCR) analysis

Total RNA was extracted from root, stem, leaf, flower, and fruit tissues of wild type Micro-Tom plants as described previously (Lee et al., 2026). Vegetative tissues and flowers were collected from 7-week-old plants, and fruits were harvested at the six ripening stages described above. RNA concentration and purity were determined using a NanoDrop 1000 spectrophotometer (Thermo Fisher Scientific, USA), and residual genomic DNA was removed by DNase I treatment. First-strand cDNA was synthesized from 3 μg of total RNA using SuperScript III Reverse Transcriptase (Invitrogen, USA) according to the manufacturer’s instructions. RT-qPCR was performed on a CFX96 Touch Real-Time PCR Detection System (Bio-Rad, USA) using iQ SYBR Green Supermix (Bio-Rad, USA) in a final reaction volume of 20 μL. The thermal cycling conditions were 95 °C for 3 min, followed by 40 cycles of 95 °C for 15 s and 60 °C for 30 s. Melting-curve analysis was performed from 65 °C to 95 °C to confirm amplification specificity. *SlALS1 (Solyc06g059880.2)* and *SlALS2 (Solyc03g044330.1)* were used as internal reference genes, and relative transcript levels were calculated using the 2^−ΔΔCt^ method with the geometric mean of the two reference genes for normalization. Three biological replicates, each analyzed in three technical replicates, were used for each sample.

### Imazethapyr treatment and sample collection

The ALS inhibitor imazethapyr (MedChemExpress, USA) was applied through the leaf vascular system from the flowering stage until fruit ripening. Imazethapyr was prepared at a final concentration of 1 μM and administered at 1 mL per plant for a total of three applications at regular intervals. The solution was injected along the leaf midrib to facilitate systemic transport throughout the plant.

Throughout the treatment period, plants were maintained under identical growth conditions. Fruits were harvested at the red-ripe stage, and carbohydrate contents were analyzed as described above. Fruit soluble solids content was measured using a digital refractometer.

## Results

### Changes in carbohydrates and amino acids during wild-type tomato fruit ripening

Wild-type Micro-Tom tomato fruits were classified according to developmental stages as previously described (**Figure 1A**) (Kanayama, 2017), and sugar accumulation patterns were analyzed throughout fruit development (**Figure 1B**). Sucrose accumulation gradually increased during fruit development and reached a maximum concentration of 0.66 mg mL^-^¹ at the turning stage (**Figure 1B**). In contrast, glucose and fructose accumulated at substantially higher levels than sucrose throughout fruit development, with average concentrations of approximately 13 mg mL^-^¹ and 18 mg mL^-^¹, respectively (**Figure 1B**).

**Figure 1:**
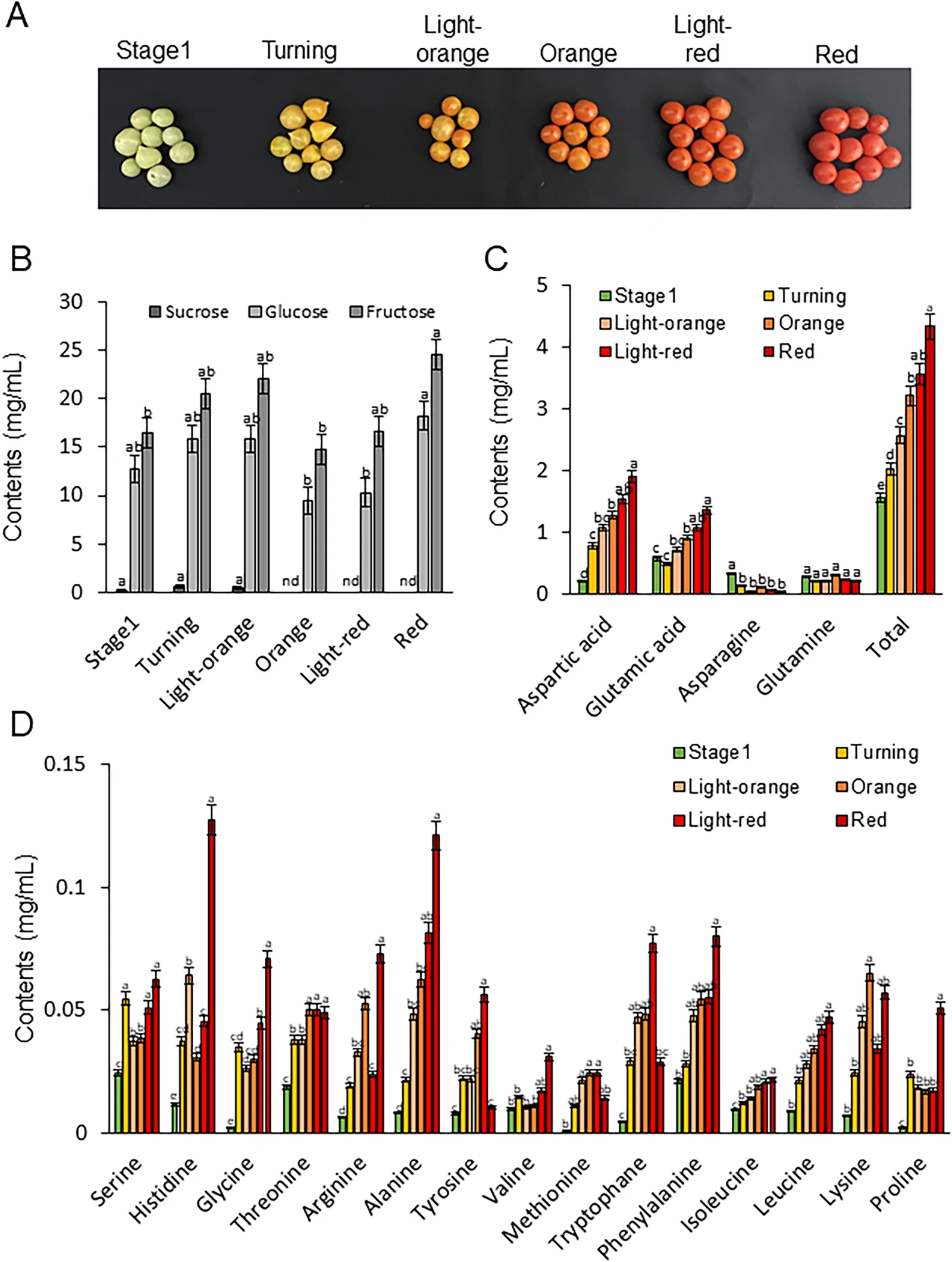
The level of carbohydrates and constituent amino acids during the ripening of wild-type tomato fruit. **(A)** Color changes of Micro-Tom tomatoes at distinct ripening stages. Fruits were classified into six distinct stages: Stage 1, Turning, Light-orange, Orange, Light-red, and Red, and representative fruits from each stage were collected and compared. **(B)** The contents of carbohydrates (sucrose, glucose, and fructose) in Micro-Tom tomato fruits were measured using HPLC. Fruits were collected from three independent plants at each ripening stage. Five to six fruits from each plant were analyzed individually, and the mean value for each plant was used as one biological replicate (n=3). Different letters above the bars indicate significant differences among the six ripening stages (Stage 1 to Red) for each sugar separately (p < 0.05), as determined by one-way analysis of variance (ANOVA) followed by Tukey’s multiple comparisons test. **(C, D)** Changes in amino acid contents during Micro-Tom tomato fruit ripening. **(C)** Main constituent amino acids and **(D)** individual amino acid levels were measured across six ripening stages. Different letters (a–f) indicate significant differences among ripening stages (p < 0.05), as determined by one-way analysis of variance (ANOVA) followed by Tukey’s multiple comparisons test. Error bars represent the standard deviation (SD) of three biological replicates.

Glucose content increased progressively from Stage 1 to the light-orange stage, followed by a transient decrease at the orange stage. Thereafter, glucose levels increased again and reached their highest level of approximately 19 mg mL^-^¹ at the red stage. Fructose content continuously increased throughout fruit ripening and reached approximately 25 mg mL^-^¹ at the red stage, remaining consistently higher than glucose levels (**Figure 1B**). In contrast, sucrose levels declined to nearly undetectable levels after the orange stage, indicating that glucose and fructose became the predominant soluble sugars during the later stages of fruit ripening (Quinet et al., 2019). During the transition from the light-red to red stage, glucose and fructose contents increased by approximately 70% and 50% compared to the stage 1, respectively, indicating intensive sugar accumulation during the final stages of fruit ripening.

Analysis of amino acid profiles across developmental stages revealed substantial metabolic reprogramming during tomato fruit ripening (**Figures 1C, D**). Similar to sugar accumulation, most amino acids exhibited a marked increase from the turning stage to the orange and red stages (**Figure 1D**). In contrast, several amino acids, including asparagine and glutamine, decreased or remained unchanged after the turning stage (**Figure 1C**).

Notably, phenylalanine, methionine, asparagine, and glutamine are known precursors of volatile compounds that contribute to tomato aroma formation (Distefano et al., 2022). Their decreased accumulation during fruit ripening may reflect increased metabolic flux toward the biosynthesis of aroma-related volatile compounds.

The BCAAs leucine, isoleucine, and valine, whose biosynthesis is initiated by acetolactate synthase (ALS), exhibited continuous increases during fruit ripening (**Figure 1D**). Although their absolute levels were lower than those of several other amino acids, all three BCAAs accumulated progressively as fruits matured. BCAAs have been reported to contribute to energy metabolism and the biosynthesis of aroma-associated volatiles in ripened fruits (Martina et al., 2021), suggesting enhanced BCAA metabolism during tomato fruit ripening.

Glutamate and aspartate, two amino acids closely associated with umami taste (Servant and Frerot, 2021; Mouritsen et al., 2019), also increased as fruit ripening progressed (**Figure 1C**). In fully ripened red-stage fruits, aspartate accounted for approximately 60% of the total amino acid pool, whereas glutamate represented approximately 20%, making it the second most abundant amino acid. These results indicate that aspartate and glutamate are the predominant amino acids accumulated during tomato fruit ripening and may contribute substantially to the characteristic flavor of mature tomato fruits.

### Expression patterns of *SlALS1* and *SlALS2* in tomato

To investigate the potential roles of *SlALS1* and *SlALS2* as key enzymes involved in BCAA biosynthesis during fruit development and ripening (**Figure 2A**), their transcript levels were analyzed in different tissues and fruit developmental stages of wild-type Micro-Tom plants (**Figures 2B–E**). As shown in **Figure 2A**, *acetolactate synthase (ALS)* catalyzes the first committed step of BCAA biosynthesis using pyruvate as a substrate, contributing to the biosynthesis of valine, leucine, and isoleucine.

**Figure 2:**
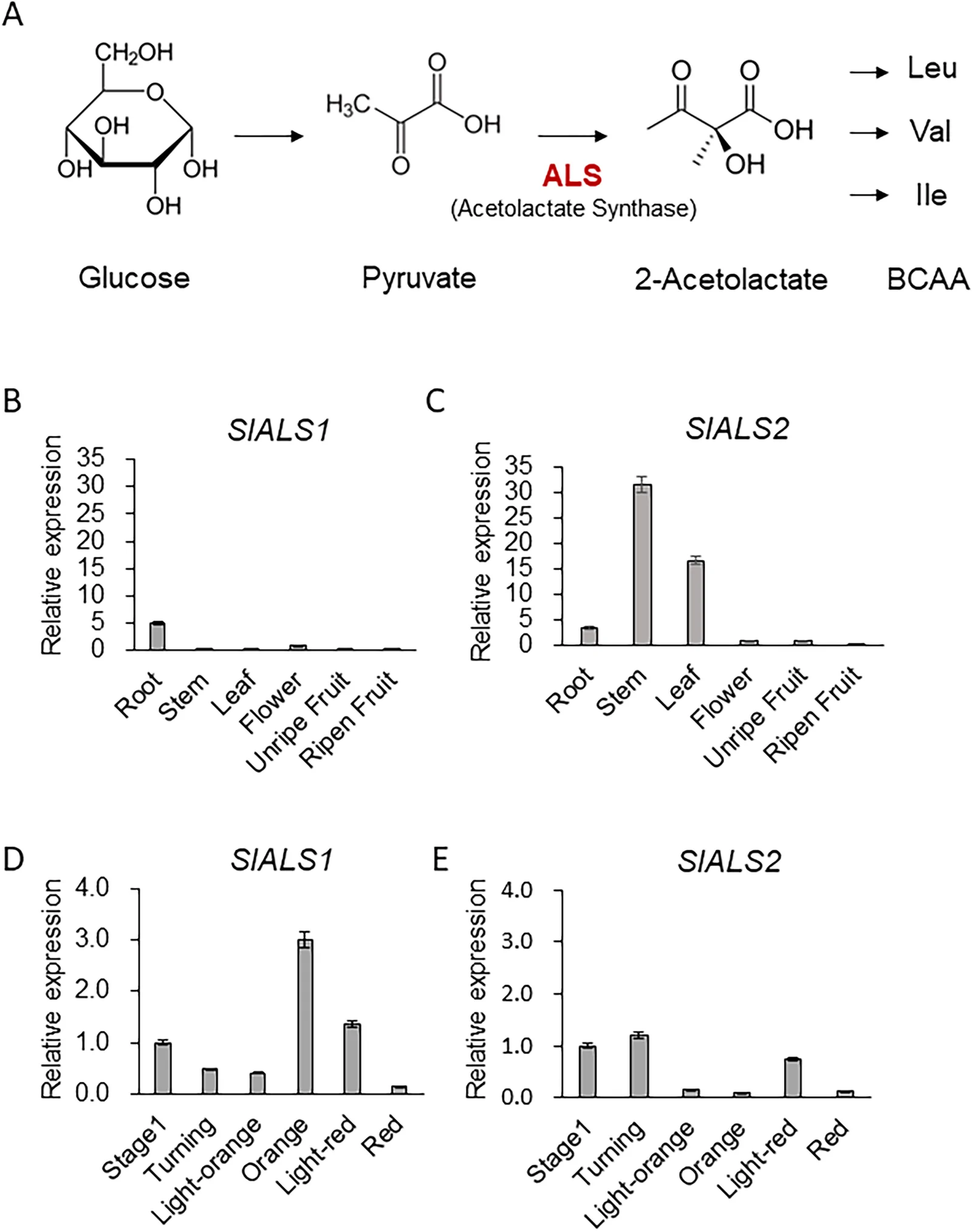
Schematic representation of the ALS-mediated BCAA biosynthetic pathway and expression profiles of SlALS1 and SlALS2 during fruit development. **(A)** A schematic diagram showing the role of acetolactate synthase (ALS) in BCAA biosynthesis, with pyruvate serving as the substrate for the ALScatalyzed reaction. **(B–E)** Spatiotemporal expression analysis of SlALS1 and SlALS2 using RT-qPCR. Relative expression levels of SlALS1. The chemical images for Glucose, Pyruvic Acid and 2-Acetolactate reproduced from Wikimedia Commons, licensed under Public Domain. **(B)** and SlALS2 **(C)** in various tissues (root, stem, leaf, flower, unripe fruit, and ripe fruit), and at different fruit ripening stages **(D, E)**. Error bars represent the standard deviation (SD) of three independent biological replicates.

*SlALS2* exhibited substantially higher expression levels than *SlALS1* in stems and leaves, with transcript abundance approximately 10–20-fold greater in these vegetative tissues (**Figures 2B, C**). In contrast, *SlALS1* showed relatively higher expression in roots (**Figure 2B**). In reproductive tissues, including flowers and fruits, *SlALS1* and *SlALS2* displayed comparable expression levels (**Figure 2C**).

During fruit ripening, *SlALS1* expression gradually increased and reached its highest level at the orange stage (**Figure 2D**), whereas *SlALS2* expression peaked earlier at the turning stage (**Figure 2E**). These distinct expression patterns suggest differential roles of the two *ALS* genes during fruit development and ripening. In particular, the increased expression of *SlALS1* during the mid-to-late stages of fruit ripening suggests that *SlALS1* may contribute to BCAA biosynthesis during fruit maturation.

### Design of gRNAs targeting tomato ALS genes

To investigate the roles of *ALS* genes in sugar and amino acid accumulation in tomato fruits, CRISPR/Cas9-mediated genome editing was employed to generate mutations in the *Acetolactate synthase* genes (**Figure 3**). Two target genes, *SlALS1* and *SlALS2* were selected, and gene-specific guide RNAs (gRNAs) were designed using the CRISPR-P v2.0 program based on their genomic sequences (**Figure 3B**).

**Figure 3:**
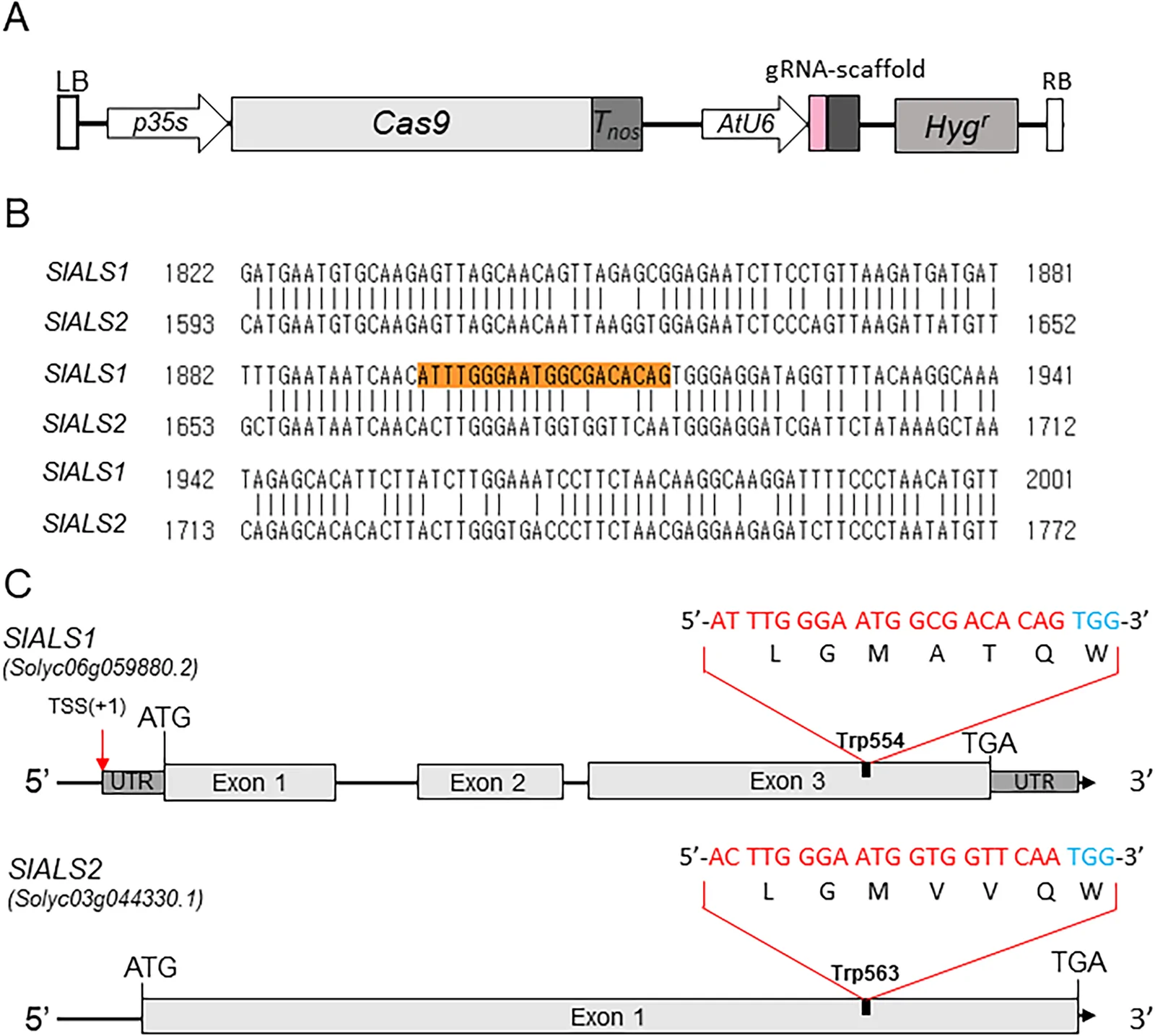
Construction map for targeted mutagenesis of the ALS genes via CRISPR/Cas9. **(A)** The schematic description of the CRISPR/Cas9-gRNA expression cassette. Cas9 expression is controlled by a CaMV 35S promoter, and the gRNA is expressed by the AtU6 promoter. **(B)** Alignment of *SlALS1* and *SlALS2* genomic sequences. The gRNA target site of *SlALS1* showing six mismatches with *SlALS2* is highlighted in orange. **(C)** Schematic diagrams of the *SlALS1* and *SlALS2* gene structures showing the gRNA target sites. Gray boxes represent exons, black lines represent introns, and gray narrow boxes indicate untranslated regions (UTRs). The transcription start site (TSS) is indicated by a red arrow. Red letters indicate the 20-bp gRNA protospacer sequences, and blue letters indicate the PAM (NGG) sequences. The gRNA target sites are located adjacent to the conserved tryptophan residues W554 in *SlALS1* and W563 in *SlALS2*. The UTR of SlALS2 has not been identified yet.

The selected gRNAs were designed to minimize potential cross targeting between *SlALS1* and *SlALS2.* Although the two genes share high sequence similarity, the target regions contained six mismatched nucleotides within the 20-bp protospacer sequence (**Figure 3B**), thereby ensuring target specificity. The target sites corresponded to amino acid positions 554 and 563 in *SlALS1* and *SlALS*2, respectively, and were located within a functionally important conserved region containing a tryptophan (W) residue. Mutations at this position have been reported to alter ALS function through amino acid substitution.

The GC contents of the selected gRNAs were 50% for *SlALS1* and 45% for *SlALS2*, which fall within the optimal range for efficient CRISPR/Cas9-mediated genome editing. Gene structure analysis revealed that the *SlALS1* target site was located within the third exon, whereas the *SlALS2* target site was located within the first exon because *SlALS2* lacks introns within its coding region (**Figure 3C**).

Based on these target designs, CRISPR/Cas9 constructs (**Figure 3A**) were generated to produce *SlALS1* and *SlALS2* knockout mutants, which were subsequently used to investigate the effects of ALS disruption on amino acid and carbohydrate metabolism in tomato fruits.

### Generation of CRISPR/Cas9-edited tomato plants targeting ALS genes

Based on the tissue specific expression profiles of *SlALS1* and *SlALS2*, *SlALS1* was expected to have a relatively smaller contribution to BCAA biosynthesis than *SlALS2* in tomato. Therefore, loss of *SlALS1* function was not expected to severely affect plant viability. In contrast, *SlALS2* exhibited high expression levels throughout vegetative tissues, suggesting a more prominent role in plant growth and development.

To generate targeted mutations, gene-specific gRNAs were designed for *SlALS1* and *SlALS2* and introduced into tomato using the CRISPR/Cas9 system (Yang et al., 2022). The growth and developmental progression of the ALS1-KO transgenic plants were evaluated under laboratory growth conditions. Representative images of ALS1-KO plants at sequential developmental stages, including vegetative growth and flowering demonstrated normal plant establishment and development following genome editing (**Figure 4A**).

**Figure 4:**
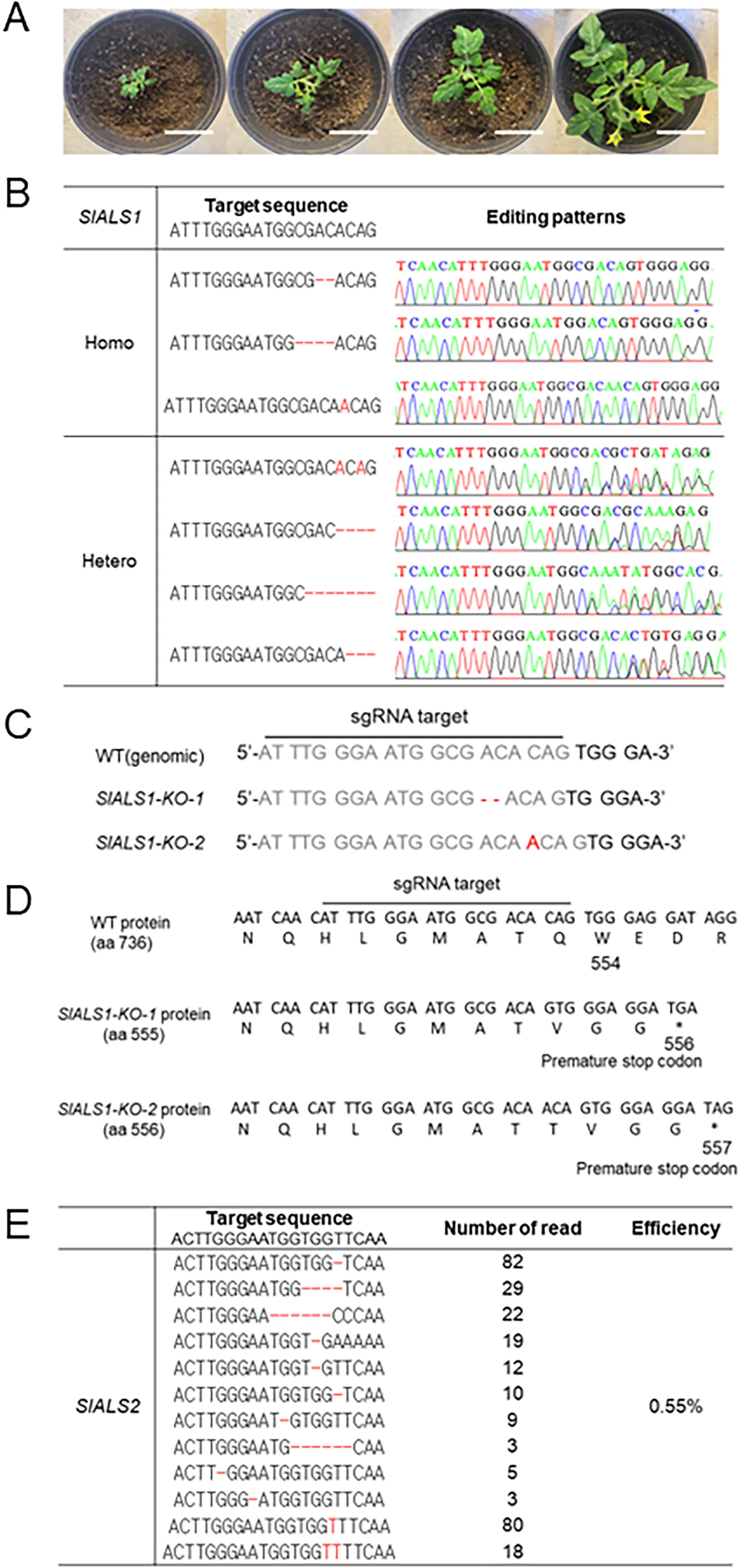
CRISPR/Cas9–mediated genome editing of *SlALS1* and *SlALS2* in tomato via stable transformation and transient expression. **(A)** Representative growth progression of ALS1-KO-1 transgenic tomato plants under greenhouse growth conditions. Images were collected at different developmental stages, including vegetative growth, flowering, and fruit development stages, to evaluate plant establishment and phenotypic stability after genome editing. Scale bars represent 5 cm and are provided as a reference for plant size at each developmental stage. **(B)** Sequence analysis of the SlALS1 target site in homozygous and heterozygous tomato mutants. In SlALS1-KO-1, which was used for further analysis, a two-base deletion was detected. The other mutant alleles contained either a four-base deletion or a single-base insertion. **(C)** Target sequence comparison of the WT, SlALS1-KO-1, and SlALS1-KO-2 alleles, showing the 2-bp deletion in SlALS1-KO-1 and the 1-bp insertion in SlALS1-KO-2. **(D)** Predicted amino acid changes resulting from the frameshift mutations in SlALS1-KO-1 and SlALS1-KO-2. **(E)** Representative editing patterns at the SlALS2 target site detected by next-generation sequencing (NGS) following transient expression of the CRISPR-Cas9 system. The number of reads (# Reads) indicates the number of sequencing reads corresponding to each detected allele. Editing efficiency represents the percentage of sequencing reads containing insertions or deletions (indels) at the target site relative to the total number of aligned reads.

Multiple homozygous mutant lines carrying edits in the *SlALS1* gene were successfully obtained (**Figure 4B**). Sequence analysis of T3-generation plants identified two distinct mutant alleles, including ALS1-KO-1 carrying a 2-bp deletion and ALS1-KO-2 carrying a 1-bp insertion within the target region. ALS1-KO-1 and ALS1-KO-2 were derived from two different T0 transgenic plants and were therefore obtained from independent transformation events. Both mutations resulted in frameshifts and were predicted to introduce premature stop codons. Specifically, the 2-bp deletion in ALS1-KO-1 caused a frameshift and introduced a premature stop codon at codon 556, resulting in a predicted truncated protein of 555 amino acids, whereas the 1-bp insertion in ALS1-KO-2 caused a frameshift and introduced a premature stop codon at codon 557, resulting in a predicted truncated protein of 556 amino acids (**Figures 4C, D**). Among these, ALS1-KO-1 was selected for subsequent phenotypic and metabolic analyses. Additional homozygous *SlALS1* mutant lines showed comparable growth and fruit developmental characteristics during preliminary evaluation. However, ALS1-KO-1 was selected for detailed metabolite profiling because its 2-bp deletion resulted in a clear frameshift mutation and predicted premature termination of translation. The predicted amino acid sequence of ALS1-KO-1 differed from that of the wild-type protein downstream of the deletion and translation was predicted to terminate at codon 556, resulting in loss of the C-terminal portion of SlALS1. Similarly, ALS1-KO-2 was predicted to produce a truncated protein of 556 amino acids due to premature termination at codon 557. These sequence changes support the classification of ALS1-KO-1 and ALS1-KO-2 as predicted loss-of-function alleles.

To evaluate the editing activity of the *SlALS2*-targeting gRNA, transient expression assays were performed in tomato cotyledons using Agrobacterium-mediated delivery. Next-generation sequencing (NGS) analysis revealed various editing events at the target site (**Figure 4E**). Most mutations occurred approximately 3–4 bp upstream of the PAM sequence, consistent with the typical indel patterns generated by SpCas9-mediated cleavage.

In contrast to *SlALS1*, homozygous *SlALS2* mutant plants were not recovered in the T1 generation. Three independent stable transformation experiments were performed, resulting in 20 independent T0 transgenic events. Approximately 100 of T1 progeny derived from 20 of T0 independent transgenic lines were screened for *SlALS2* mutations. Heterozygous *SlALS2* mutant plants were identified. however, no homozygous or biallelic mutant plants were found in the T1 generation. Given these results, we acknowledge that the absence of homozygous or biallelic *SlALS2* mutants does not enough to demonstrate that *SlALS2* is essential for tomato growth and development. Nevertheless, these observations raise the possibility that *SlALS2* may contribute to plant growth and development, although this potential role remains to be experimentally validated.

### Altered amino acid, carbohydrate, and organic acid metabolism in SlALS1-KO tomato fruits

To investigate the effects of SlALS1 disruption on amino acid metabolism, amino acid profiles of SlALS1-KO and wild-type tomato fruits were compared (**Figure 5A**). The concentrations of the BCAAs valine, leucine, and isoleucine were significantly reduced in SlALS1-KO fruits relative to wild-type fruits. Specifically, valine decreased from 0.030 mg mL^-^¹ in wild-type Micro-Tom fruits to 0.022 mg mL^-^¹ in SlALS1-KO-1 fruits, while isoleucine decreased from 0.020 to 0.013 mg mL^-^¹ and leucine decreased from 0.046 to 0.028 mg mL^-^¹, corresponding to reductions of approximately 26.7%, 35.0%, and 39.1%, respectively. To determine whether this pattern was also observed in an independent SlALS1 mutant line, the levels of valine, leucine, and isoleucine were additionally examined in SlALS1-KO-2 fruits. SlALS1-KO-2 showed lower mean levels of all three BCAAs compared with wild-type fruits, with valine, isoleucine, and leucine levels of 0.0250, 0.0155, and 0.0343 mg mL^-^¹, respectively, consistent with the pattern observed in SlALS1-KO-1. In addition, the levels of glutamic acid, histidine, arginine, alanine, phenylalanine, lysine, and proline also showed a general decrease in SlALS1-KO fruits. In contrast, the concentrations of aspartic acid, serine, glycine, threonine, tyrosine, and methionine were increased.

**Figure 5:**
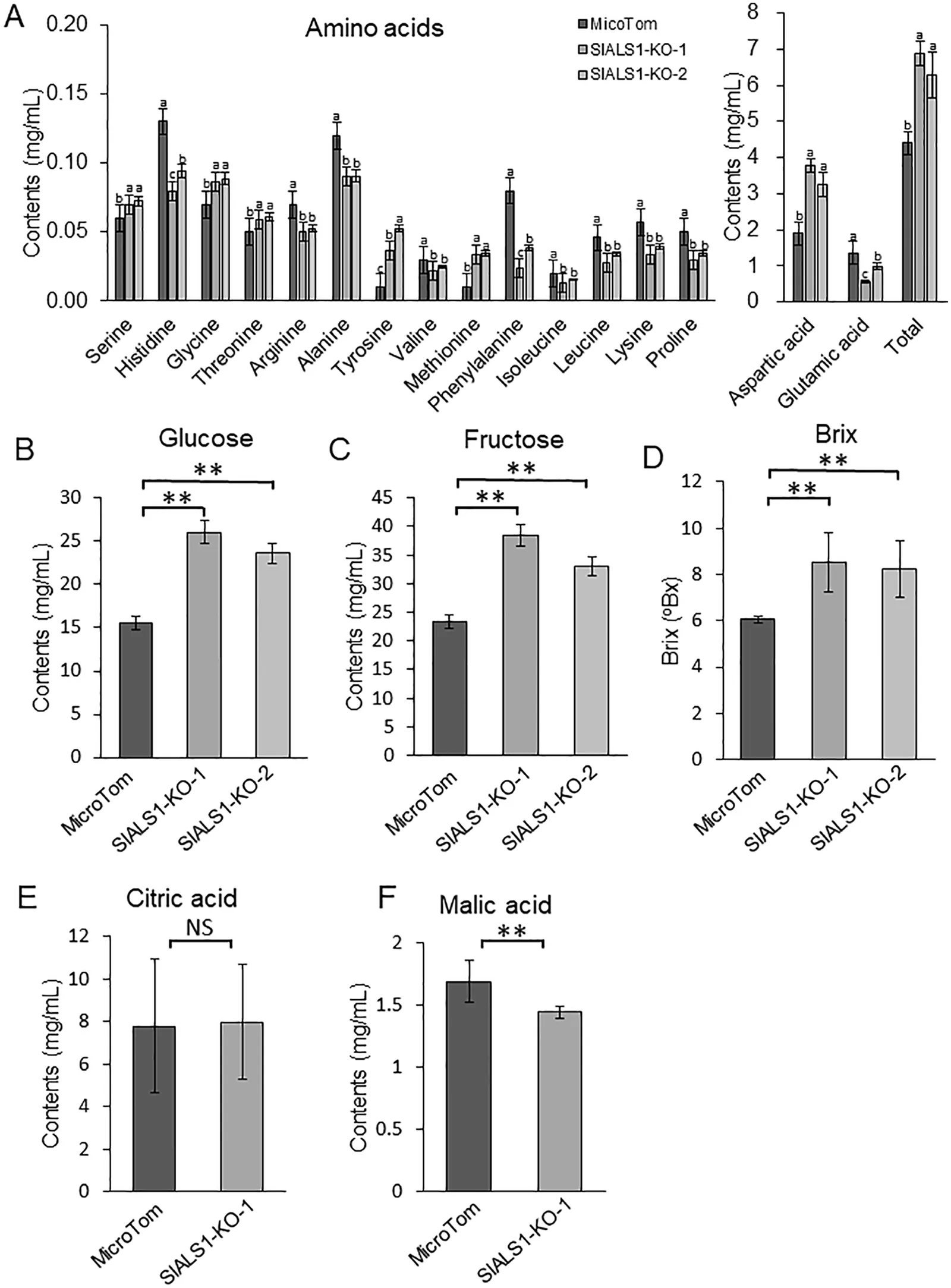
Metabolic profiling of amino acids, carbohydrates, organic acids, and soluble solids in SlALS1-KO tomato fruits. **(A)** Comparative analysis of amino acid contents among wild-type (Micro-Tom), SlALS1-KO-1, and SlALS1-KO-2 tomato fruits at the red ripening stage. Different lowercase letters indicate significant differences among genotypes (p < 0.05), as determined by one-way analysis of variance (ANOVA) followed by Tukey’s multiple comparisons test. **(B–D)** Quantification of glucose **(B)**, fructose **(C)**, and total soluble solids (Brix) **(D)** in wild-type, SlALS1-KO-1, and SlALS1-KO-2 fruits. **(E, F)** Quantification of citric acid **(E)** and malic acid **(F)** in wild-type and SlALS1-KO-1 fruits. Fruits were collected from three independent plants per genotype at the red ripening stage. Five to six fruits from each plant were analyzed individually, and the mean value calculated from each plant was considered as one biological replicate (n=3). Data are presented as mean ± standard deviation (SD). Asterisks indicate significant differences from the corresponding Micro-Tom control, as determined by Student’s t-test (p < 0.01).

Among these metabolites, aspartic acid exhibited the most pronounced increase, reaching 3.8 mg mL^-^¹ in SlALS1-KO fruits compared with 1.9 mg mL^-^¹ in wild-type Micro-Tom fruits. Although SlALS1 has been proposed to play a supplementary role to SlALS2, its quantitative contribution to BCAA biosynthesis has not been clearly demonstrated. The present study provides quantitative evidence that loss of SlALS1 function reduces BCAA accumulation in red-stage tomato fruits and alters the abundance of multiple non-BCAA amino acids. The lower mean levels of valine, leucine, and isoleucine observed in both independent SlALS1 mutant lines further support the involvement of SlALS1 in BCAA metabolism. Notably, aspartic acid is closely associated with organic acid metabolism and central carbon metabolism. Therefore, the increased accumulation of aspartic acid observed in SlALS1-KO fruits may reflect broader metabolic reprogramming associated with carbon allocation. However, further studies will be required to clarify the underlying mechanisms (Oruna-Concha et al., 2007).

Given the substantial changes in amino acid composition, carbohydrate accumulation was further examined in SlALS1-KO fruits. Sucrose was below the detection limit in both SlALS1-KO and wild-type fruits. In contrast, glucose and fructose contents were significantly increased in SlALS1-KO-1 fruits by approximately 30% and 37%, respectively, compared with wild-type Micro-Tom fruits (**Figures 5B, C**).

Consistent with these changes in soluble sugar content, SlALS1-KO-1 fruits exhibited a significantly higher soluble solids content (Brix) than wild-type fruits (**Figure 5D**). The average Brix value of SlALS1-KO-1 fruits was 9.4, representing a 46.9% increase relative to the wild-type value of 6.4. Although Brix reflects total soluble solids and may include contributions from organic acids and other soluble metabolites, the observed increase was accompanied by substantial elevations in glucose and fructose levels. Similar increases in glucose, fructose, and soluble solids (Brix) were also observed in the independent SlALS1-KO-2 line (**Figures 5B–D**), indicating that the enhanced sugar accumulation is reproducible and associated with SlALS1 disruption rather than being unique to a single edited line.

To determine whether changes in organic acid metabolism contributed to fruit taste characteristics, the major organic acids, citric acid and malic acid, were quantified (**Figures 5E, F**). Citric acid content did not differ significantly between SlALS1-KO and wild-type fruits, indicating that the major source of fruit acidity remained largely unchanged. In contrast, malic acid content was significantly lower in SlALS1-KO fruits than in wild-type fruits. The reduction in malic acid, together with the increased accumulation of glucose and fructose, suggests an increase in the ratio of sugars to organic acids, which is an important determinant of fruit taste and perceived sweetness.

Taken together, these results indicate that disruption of SlALS1 reduces BCAA accumulation while promoting sugar accumulation in tomato fruits. Importantly, the independent SlALS1-KO-2 line showed lower mean levels of valine, leucine, and isoleucine together with increased glucose, fructose, and soluble solids contents, consistent with the metabolic phenotype observed in SlALS1-KO-1. Thus, the concurrent pattern of lower BCAA levels and increased sugar accumulation observed in two independent SlALS1 mutant lines supports an association between SlALS1-mediated BCAA metabolism and carbohydrate accumulation in tomato fruits. In the representative SlALS1-KO-1 line, SlALS1 disruption was also associated with reduced malic acid levels without significant changes in citric acid content, indicating altered carbon metabolism and changes in the balance between sugars and organic acids in tomato fruits. These findings are consistent with previous observations that suppression of BCAA biosynthesis can influence carbon partitioning and enhance sugar accumulation.

### Effects of ALS inhibition on sugar accumulation in tomato fruits

Previous studies have reported that treatment with herbicides that inhibit amino acid biosynthesis, such as glyphosate, can induce carbon metabolic reprogramming and increase sugar accumulation in plant tissues (Li et al., 2019; Zulet-Gonzalez et al., 2023; de Almeida Silva et al., 2022). For example, glyphosate treatment has been shown to increase soluble sugar levels and alter central carbon metabolism in soybean and *Amaranthus* leaves (Li et al., 2019; Zulet-Gonzalez et al., 2023). In sugarcane, low dose glyphosate treatment was reported to enhance sucrose accumulation and promote ripening (de Almeida Silva et al., 2022).

To further investigate the relationship between ALS inhibition and sugar accumulation, the ALS inhibitor imazethapyr was applied to tomato plants during fruit ripening, and carbohydrate levels were subsequently analyzed in red-stage fruits. Fruits harvested from plants treated with imazethapyr exhibited markedly higher glucose and fructose contents than untreated controls (**Figures 6A, B**). Glucose and fructose concentrations reached approximately 40 mg mL^-^¹ and 38 mg mL^-^¹, respectively, whereas both sugars were present at approximately 23 mg mL^-^¹ in untreated fruits.

**Figure 6:**
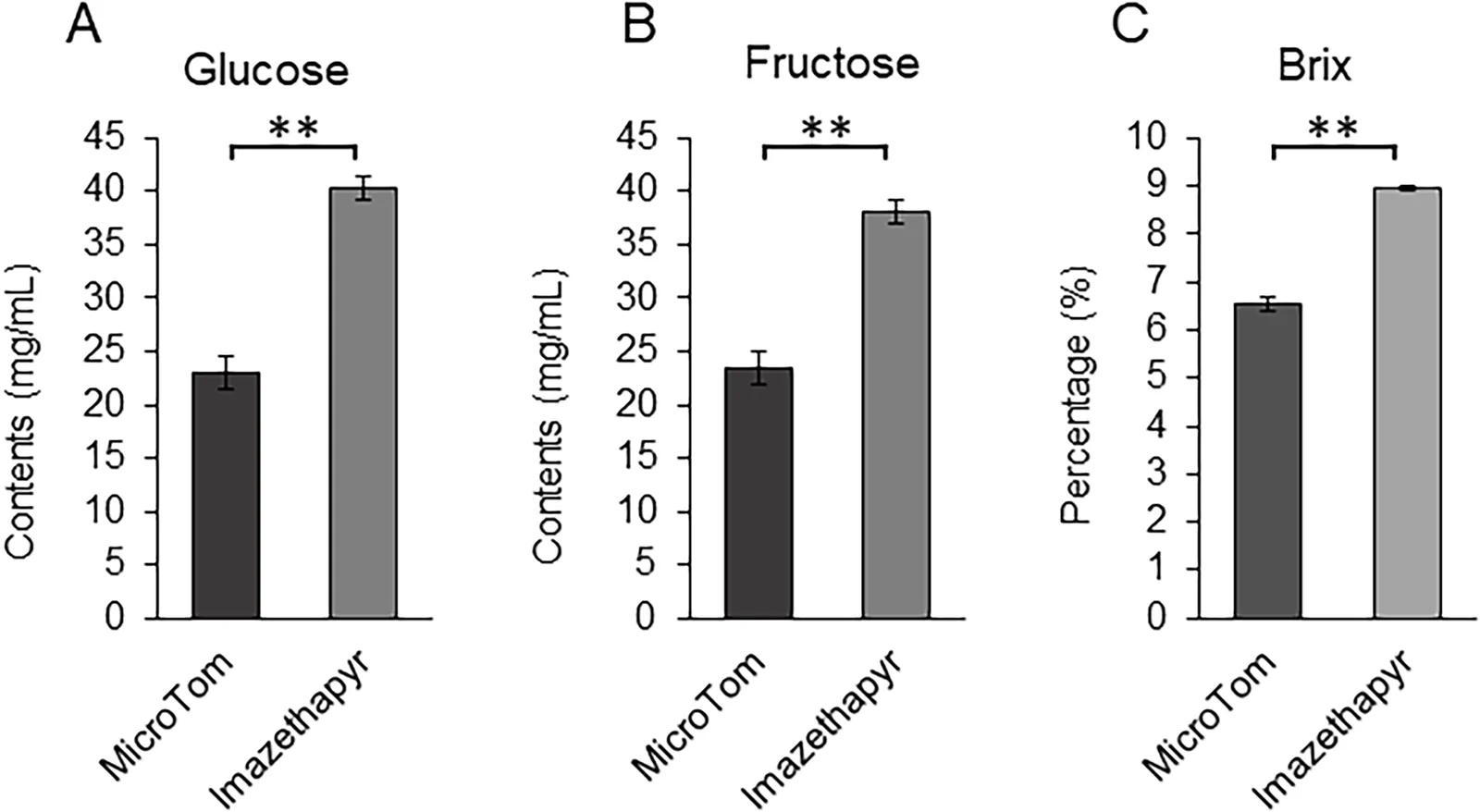
Effects of the ALS inhibitor imazethapyr on carbohydrate metabolism in wild-type tomato fruits. Imazethapyr was applied from the flowering stage until the fruit ripening stage to evaluate the impact of ALS inhibition. **(A)** Glucose and **(B)** fructose concentrations, and **(C)** total soluble solids (Brix) were quantified in fruits at the red ripening stage. Data are presented as mean ± standard deviation (SD) of biological replicates (n=5). Asterisks indicate significant differences between the control and imazethapyr-treated groups, as determined by Student’s t-test (p < 0.01).

Consistent with the increased sugar content, soluble solids content was also significantly elevated following imazethapyr treatment (**Figure 6C**). The average Brix value was 9.0 in fruits from imazethapyr treated plants, compared to 6.5 in untreated wild-type fruits.

These results demonstrate that chemical inhibition of ALS promotes sugar accumulation in tomato fruits, consistent with the metabolic changes observed in SlALS1-KO plants. The increased glucose, fructose, and Brix values observed following imazethapyr treatment further support a relationship between ALS activity and carbohydrate metabolism during tomato fruit ripening (Zabalza et al., 2013).

## Discussion

In this study, we used the CRISPR/Cas9 system to edit members of the tomato *ALS* gene family (*SlALS1* and *SlALS2*) and investigated the resulting changes in carbohydrate and amino acid metabolism. Our results demonstrated that disruption of *SlALS1* markedly promoted glucose and fructose accumulation in tomato fruits while simultaneously altering amino acid composition. These findings, obtained from the ALS1-KO knockout line, indicate that the ALS pathway is involved not only in BCAA biosynthesis but also in the regulation of carbohydrate allocation and metabolic flux during fruit ripening.

Comparison of the expression patterns of *SlALS1* and *SlALS2* suggested functional divergence between the two genes. *SlALS2* was highly expressed in vegetative tissues, including leaves, stems, and immature fruits, indicating a potentially important role during plant growth and development, consistent with previous reports describing the importance of ALS genes in plant development (Wang et al., 2021). Although homozygous *SlALS2* knockout plants were not recovered among approximately 100 T1 progeny derived from 20 independent T0 transformation events, this observation alone does not establish that *SlALS2* is required for normal vegetative growth. Thus, the potential role of *SlALS2* in plant growth and development remains a hypothesis requiring further investigation. Conversely, homozygous *SlALS1* knockout plants were readily obtained, suggesting that loss of *SlALS1* function is compatible with plant viability under the tested growth conditions.

One of the most notable findings of this study was the significant increase in glucose and fructose contents in SlALS1-KO fruits. This observation is consistent with previous studies reporting that inhibition of ALS activity can induce carbon metabolic reprogramming and promote sugar accumulation in *Arabidopsis thaliana* (Qian et al., 2011). Similar increases in soluble sugars have been observed following treatment with herbicides that inhibit ALS activity in several crop species (Kumar et al., 2025). Consistently, application of the ALS inhibitor imazethapyr also resulted in elevated sugar accumulation in tomato fruits in the present study.

When ALS activity is reduced, carbon flux from pyruvate into the BCAA biosynthetic pathway is expected to decrease. Consequently, carbon resources may be redirected toward alternative metabolic pathways, including soluble sugar accumulation (Paul and Pellny, 2003). In this study, the observed increase in glucose and fructose levels in SlALS1-KO fruits points toward a potential shift in sugar metabolism, possibly associated with altered sucrose cleavage and/or hexose metabolism, although further biochemical analyses will be required to verify this hypothesis. Taken together, these observations suggest that SlALS1 disruption affects not only amino acid biosynthesis but also broader carbon–nitrogen metabolic balance, thereby influencing carbohydrate metabolism during fruit ripening.

The asymmetric changes observed in amino acid composition further support the occurrence of metabolic reprogramming in SlALS1-KO fruits. The reductions in valine, leucine, and isoleucine were expected consequences of impaired ALS-mediated BCAA biosynthesis and are consistent with previous studies in *Arabidopsis* and maize reporting decreased BCAA levels following inhibition of the ALS/AHAS pathway. In contrast, the substantial increase in aspartate may be associated with an amino acid starvation response (Han et al., 2021; Yang et al., 2020). Under conditions in which the synthesis of specific amino acids is restricted, plants often reconfigure nitrogen and carbon metabolism to maintain metabolic homeostasis. Aspartate serves as a central precursor for multiple amino acids and nitrogen-containing metabolites and plays an important role in maintaining the balance between carbon and nitrogen metabolism under stress conditions (Han et al., 2021). Therefore, the increased accumulation of aspartate observed in SlALS1-KO fruits may represent a compensatory metabolic response to disrupted BCAA biosynthesis (Galili, 2011).

Importantly, genome editing of *SlALS1* significantly increased fruit soluble solids content. The average Brix value of SlALS1-KO fruits reached 9.4, substantially higher than that of wild-type Micro-Tom fruits. Previously identified tomato sweetness-related genes have largely been associated with sucrose transport, sucrose cleavage, or starch metabolism. For example, *LIN5*, which encodes a cell wall invertase, promotes the conversion of sucrose into glucose and fructose, thereby increasing hexose accumulation (Fridman et al., 2004). Similarly, genes encoding AGPase, SPS, SWEET transporters, and SUT transporters have been reported to influence fruit sugar content through regulation of starch metabolism, sucrose biosynthesis, and sugar transport over long distances (Braun et al., 2014; Osorio et al., 2014).

In addition to sugar accumulation, fruit taste is strongly influenced by the balance between sugars and organic acids. Citric acid and malic acid are the predominant organic acids in tomato fruits and are major determinants of fruit acidity. In the present study, citric acid content did not differ significantly between SlALS1-KO and wild-type fruits, whereas malic acid content was significantly reduced in SlALS1-KO fruits. The combination of increased glucose and fructose contents with reduced malic acid suggests an increased ratio of sugars to organic acids, which may be associated with enhanced sweetness potential in SlALS1-KO fruits. These findings indicate that disruption of SlALS1 influences not only carbohydrate accumulation but also the balance between sugars and organic acids, thereby potentially contributing to improved fruit sweetness.

Unlike these genes previously characterized for their roles in sugar metabolism, *SlALS1* appears to influence sugar accumulation indirectly through modulation of BCAA biosynthesis and carbon allocation. Reduced *SlALS1* activity limits BCAA biosynthesis and may alter the allocation of carbon skeletons between amino acid and carbohydrate metabolism. This metabolic rebalancing could contribute to increased hexose accumulation. The observation that glucose and fructose increased without corresponding changes in sucrose concentration is consistent with the possibility that *SlALS1* disruption preferentially affects hexose accumulation rather than overall sucrose levels.

Collectively, these findings suggest that SlALS1 represents a potential regulator of fruit sugar accumulation through modulation of amino acid biosynthesis and carbon–nitrogen metabolic balance. Although targeted disruption of SlALS1 increased fruit sugar content, additional evaluation of agronomic traits, including plant growth, fruit yield, and fruit quality under diverse growing conditions, will be necessary to determine its suitability as a target for tomato breeding. In conclusion, this study demonstrates that although SlALS1 is not required for plant viability under the tested growth conditions, it functions as an important metabolic regulator associated with amino acid and carbohydrate accumulation in tomato fruits. CRISPR/Cas9-mediated disruption of SlALS1 increased fruit sugar content and provides new insights into the relationship between BCAA biosynthesis and carbohydrate metabolism during tomato fruit ripening. These findings suggest that SlALS1 may represent a potential target for improving fruit quality, however, further evaluation of agronomic traits, including plant growth, fruit yield, flowering time, ripening characteristics, and field performance, will be required before its practical application in tomato breeding can be considered.

## Concluding remarks

This study demonstrates that SlALS1 functions as an important metabolic regulator linking BCAA biosynthesis with carbohydrate metabolism during tomato fruit ripening. CRISPR/Cas9-mediated disruption of SlALS1 significantly reduced the accumulation of BCAAs while promoting glucose and fructose accumulation, resulting in a substantial increase in fruit soluble solids content (Brix). Similar metabolic responses induced by the ALS inhibitor imazethapyr further support the role of ALS activity in regulating carbon allocation and sugar accumulation. Unlike previously reported sugar-related genes that primarily regulate sucrose transport or metabolism, SlALS1 appears to influence fruit sweetness indirectly by modulating carbon partitioning between amino acid biosynthesis and carbohydrate accumulation. These findings identify SlALS1 as a previously unrecognized target for improving tomato fruit quality through precision genome editing. Overall, this work expands our understanding of the relationship between amino acid metabolism and fruit sugar accumulation and provides a promising strategy for developing high-sugar tomato cultivars. Future studies evaluating fruit quality traits, volatile compounds, agronomic performance, and field stability of SlALS1-edited tomatoes will facilitate the practical application of this metabolic engineering approach in tomato breeding.

## Data Availability

The raw data supporting the conclusions of this article will be made available by the authors, without undue reservation.
